# Dually Acting Nonclassical 1,4-Dihydropyridines Promote the Anti-Tuberculosis (Tb) Activities of Clofazimine

**DOI:** 10.3390/molecules24162873

**Published:** 2019-08-08

**Authors:** Fabian Lentz, Norbert Reiling, Gabriella Spengler, Annamária Kincses, Andrea Csonka, Joseph Molnár, Andreas Hilgeroth

**Affiliations:** 1 Institute of Pharmacy, Martin-Luther-University Halle-Wittenberg, 06120 Halle, Germany; 2 Research Center of Borstel, Leibniz Lung Center, 23845 Borstel, Germany; 3 Department of Medical Microbiology, University of Szeged, 6720 Szeged, Hungary

**Keywords:** antibacterial activity, synthesis, substituent, structure–activity, inhibition

## Abstract

The number of effective antituberculotic drugs is strongly limited to four first-line drugs in standard therapy. In case of resistances second-line antibiotics are used with a poor efficacy and tolerability. Therefore, novel antituberculotic drugs are urgently needed. We synthesized novel nonclassical 1,4-dihydropyridines and evaluated their antituberculotic properties depending on substituent effects. Preferred substituents could be identified. As related classical 1,4-dihydropyridines are known as inhibitors of the transmembrane efflux pump ABCB1 in cancer cells, we wondered whether a use of our compounds may be of favour to enhance the antituberculotic drug efficacy of the second-line antituberculotic drug clofazimine, which is a known substrate of ABCB1 by a suggested inhibition of a corresponding efflux pump in *Mycobacterium tuberculosis* (Mtb). For this, we determined the ABCB1 inhibiting properties of our compounds in a mouse T-lymphoma cell line model and then evaluated the drug-enhancing properties of selected compounds in a co-application with clofazimine in our Mtb strain. We identified novel enhancers of clofazimine toxicity which could prevent clofazimine resistance development mediated by an efflux pump activity.

## 1. Introduction

Tuberculosis (Tb) is the ninth cause of global death and the leading one from a single infectious agent, thus ranking above human immunodeficiency virus (HIV) [1,2]. The present treatment regimen of Tb with the four drugs isoniazid, rifampicin, ethambutol and pyrazinamide as first-line antituberculostatic agents lasts for six months with a high efficacy of about 90% in the cases of the so-called drug-susceptible Tb [1,3,4]. Occurring problems with compliance, sub-optimal drug levels or tolerability lead to resistances [1]. In the case of a resistance against either isoniazid or rifampicin a drug-resistant Tb results which currently contributes about 50% of the global resistances [1]. A resistance against both isoniazid and rifampicin results in a multidrug-resistant Tb (MDR-Tb) [1]. In cases of such a resistance, second line antibiotics of the fluoroquinolone group or the injectable agents kanamycin, amikacin or capreomycin are used [5]. The therapy with those drugs is characterized by low efficacy, high toxicity, a long duration and a poor outcome of 54% [5,6]. A treatment of the so-called extensively drug-resistant Tb (MDR/XDR-Tb) which includes additional resistances against one fluoroquinolone and one-injectable agent is very difficult with an outcome of < 30% and thus MDR/XDR-Tb mainly contributes to the Tb mortality rates [1,5,6]. Therefore, there is a strong need for novel antituberculostatic drugs. 

Classical 1,4-dihydropyridines have been investigated to act as antituberculostatic agents [7]. They showed percentual inhibitions of the mycobacterial growth at a given concentration. Their mode of action was suggested to be similar to that of isoniazide (INH). However, for isoniazide several modes of actions are discussed to block the synthesis of mycolic acid as an essential part of the mycobacterial cell wall. Moreover, those classical 1,4-dihydropyridines own an unsubstituted nitrogen and electron-withdrawing substituents at the 2-position of the 4-phenyl residue. Both structural properties are a prerequisite for the compounds’ abilities to act as antihypertensive agents. So those compounds are excluded for a use as antituberculostatic agents. 

We developed novel nonclassical 1,4-dihydropyridines with a substituted nitrogen and without 2-substituents at the 4-phenyl residue to provide compounds which may exclusively act as antituberculostatic agents. Those classical 1,4-dihydropyridines are known to act as inhibitors of the transmembrane efflux pump ABCB1 in cancer cells where they enhance the toxicity of applicated cytostatic agents [8,9,10]. Therefore, we wondered whether our nonclassical 1,4-dihydropyridines may act in a similar way towards ABCB1 and whether they may increase the toxicity of antituberculostatic drugs due to an inhibition of a mycobacterial efflux pump similar to ABCB1. Furthermore drug-toxicity enhancing effects of promising compounds have been investigated.

## 2. Results and Discussion

### 2.1. Synthesis of the 1,4-Dihydropyridines

**Scheme 1:**
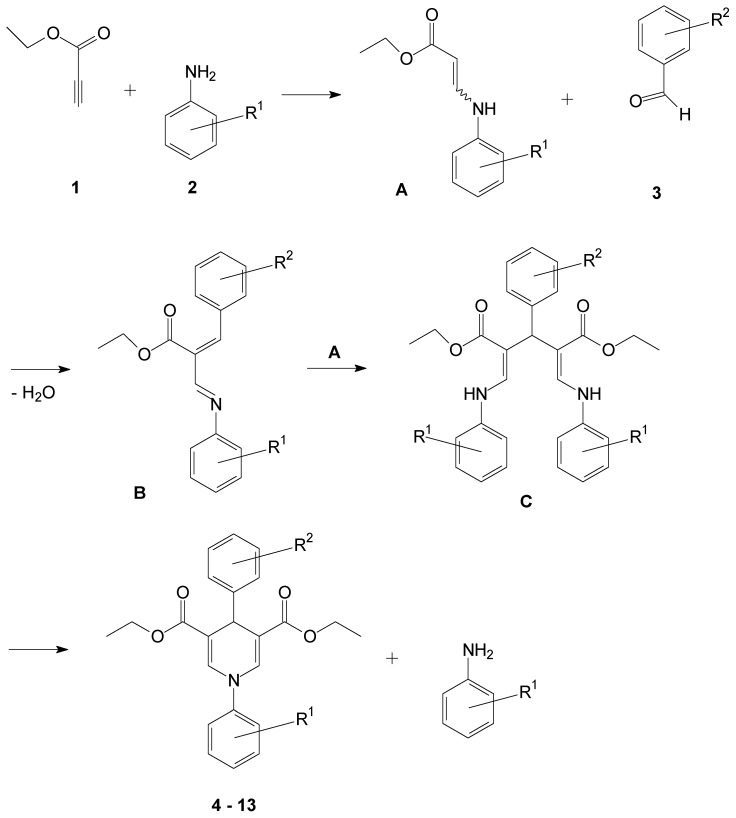
Formation of the 1,4-dihydropyridines **4**–**13** in EtOH, 3–73 h, 80 °C.

Contrasting the formation of the classical 1,4-dihydropyridines which follows the principles of the Hantzsch synthesis in ethanol or methanol depending on the nature of the used ethyl or methyl acetoacetate [11], the nonclassical 1,4-dihydropyridines without a dimethyl substitution in both the 2- and the 6-position of the 1,4-dihydropyridine scaffold are formed alternatively in a one-pot reaction by the use of an aromatic aldehyde, ethyl propiolate and an amine compound (Scheme 1). 

The mechanism followd a primary formation of the amino acrylate **A** by the reaction of one molecule ethyl propiolate **1** and the used substituted aniline compound **2** [12]. In a second reaction the aromatic aldehyde **3** condenses with the nucleophilic C-3 of amino acrylate **A** to give intermediate **B** [12,13]. In a reaction of another compound **A** and **B**, that both have been detectable in the reaction mixtures by mass spectrometry, compound **C** is formed which cyclizes under aniline elimination to give the target structure of **4**–**18** [13]. The product formation was carried out in ethanol under acetic acid catalysis using one equivalent of the aromatic aldehyde **3** and of the aniline **2** and two equivalents of ethyl propiolate **1**. The reaction proceeding was followed by thin layer chromatography (TLC) and mass spectrometry.

After finishing the reaction, the acetic acid was removed by extraction with ethyl acetate versus brine solution (10%). After drying of the organic layer, filtration and evaporation in vacuum the remaining oil was partly purified by column chromatography over silica gel and mixtures of ethyl acetate and cyclohexane to give the pure compound fractions from which the respective target structures crystallized. Spectroscopically the compounds were characterized by high-field shifts of the 4-protons of the 1,4-dihydropyridine scaffold to about 4.8 ppm and those of the 2- and 6-protons appeared as a singlet at about 7.4 ppm. 

### 2.2. Antituberculostatic Activity of the 1,4-Dihydropyridines

The antituberculostatic activity was determined using a green-fluorescent protein (GFP) expressing virulent *Mycobacterium tuberculosis* (Mtb) strain H37Rv. This allowed the monitoring of mycobacterial growth by measuring GFP-fluorescence at 528 nm. The potentially growth-inhibiting 1,4-dihydropyridines **4–18** were incubated with the mycobacteria at a concentration of 1 µg per mL and isoniazid was used as the control. The reduced measured fluorescence amounts under 1,4-dihydropyridine application as result of a growth inhibition were each related to that of the untreated control cells and the resulting percentage inhibitions of growth are given in Table 1. We investigated the effect of methyl- and chloro-substituents in the aniline residue which contribute to the lipophilic properties of the compounds. Additionally, alkoxy functions were introduced in the 4-phenyl residue to evaluate a lipophilic influence of methoxy and benzyloxy. All these substituents showed favourable effects in recent studies of the classical 1,4-dihydropyridines [7]. Amino functions for comparison were excluded as they would have interfered with the synthesis of the compounds in which the aniline nitrogen function builds the core of the 1,4-dihydropyridines. 

Compound **4** [14] with an unsubstituted 4-phenyl residue and the *N* 2-chlorophenyl substitution showed a residual antituberculostatic activity of 9%. If the *N* 2-chloro function was replaced with a 2-methyl function in compound **5** we found main increases in activity up to 34%. If the 4-phenyl residue was replaced with a 3-methoxyphenyl residue we found increases in the activity of the *N* 2-chlorophenyl derivative **6** and also the *N* 2-tolyl derivative **7** was better than that without the 3-methoxy phenyl function **5** reaching an activity of 40%. That activity was almost half of the activity of the used isoniazid (INH) control. If the 3-methoxy function of the 4-phenyl residue moved to the 4-position in compounds **8** and **9** we found decreases in activity for both either the *N* 2-chlorophenyl or the 2-tolyl substituted compounds. If two methoxy functions in the 3- and the 4-position of the 4-phenyl residue were combined in derivatives **10** and **11** we found only a residual activity for the *N* 2-chlorophenyl derivative **10** whereas the *N* 2-tolyl compound **11** was no more active. Therefore, the 3-methoxy substitution was the most favourable 4-phenyl substitution. Next, we replaced that only 3-methoxy function with a 3-benzyloxy function in compounds **12** and **13**. We found a loss of activity for the *N* 2-chlorophenyl compound **9** and a main reduction of activity for the *N* 2-tolyl compound **13** with 17%. From the first discussed data the following structure–activity relationships can be concluded: The 2-methyl group with its inductive effect was the most favourable aniline substitution contrasting the electron-withdrawing chloro-substitution. An additional 3-methoxy phenyl function with electron-pushing effects strengthened the observed activity also for the less favourable 2-chloro aniline substitution. The favourable effect of the introduced methoxy function in the 4-phenyl residue encouraged us to place it alternatively in the aniline residue. While the synthesis with the 3-methoxy aniline failed, the use of the 4-methoxy aniline was successful. We also tried to synthesize 4-phenyl derivatives with the chloro-electron withdrawing function in both the 2- and 3-position for comparison, but failed due to an insufficient aldehyde reactivity and steric reasons, also in the case of an alternative 2-methyl substitution. The only 4-methoxy aniline substituted compound **14** resulted in a main increase in bacterial growth inhibition of 60%. If that 4-methoxy aniline substitution was combined with a 3-methoxy function in the 4-phenyl residue in derivative **15** a slight decrease to 41% in the mycobacterial growth inhibition was found. If the 3-methoxy function moved to the 4-position of the 4-phenyl residue in compound **16** we found a further reduction of the mycobacterial growth inhibition to 35%. However, a combination of a 3- and 4-methoxy phenyl substitution in derivative **17** increased the growth inhibition up to 59%. A combination of the 4-methoxy aniline substitution with the 3-benzyloxy phenyl substitution in compound **18** led to a main reduction of the growth inhibition to 7%. Finally, it can be stated that the 4-methoxy function in the aniline residue with its electron-pushing effects was the best 1,4-dihydropyridine substitution. Methoxy functions placed in the 4-phenyl residue showed favourable effects with the 3-substituion being better than the 4-substitution. 

### 2.3. ABCB1 Inhibitory Activity of the 1,4-Dihydropyridines

We used a mouse T-lymphoma cell line model to evaluate the potential ABCB1 inhibitory properties of our 1,4-dihydropyridines. Within the studies we determined the amount of the ABCB1 fluorescent substrate rhodamine 123 in two types of cells: first in cells which did not express the human ABCB1 as control cells and then in cells which expressed ABCB1 after ABCB1 retrovirus gene transfer. Due to the ABCB1 expression the uptake of rhodamine was reduced in those cells. Then both cells were pre-incubated with our potential inhibitors and the fluorescence amounts of added rhodamine 123 was determined again. In the case of an ABCB1 inhibition the amounts in the ABCB1 expressing cells mainly increased due to the inhibition. Finally, a FAR (fluorescence activity ratio) value was calculated by a relation of the amounts of fluorescence uptake into the ABCB1 expressing cells under inhibitor treatment versus untreated cells and the amounts of fluorescence in the control cells under inhibitor treatment versus the untreated control cells. Therefore, compounds with FAR values > 1.1 were found active. The FAR values are shown in Table 1.

The determined ABCB1 inhibitory activity of the only 4-phenyl substituted compounds **4** and **5** were moderate with FAR values of 11.20 and 8.19, respectively, similar to that of the used standards verapamil and tariquidar. The 3-methoxy function in compounds **6** and **7** increased the activity to similar values of about 33 for both compounds. If the 4-methoxy function moved to the 4-position of the 4-phenyl residue in derivatives **8** and **9** the activity of the *N* 2-chlorophenyl compound **8** increased whereas that of the *N* 2-tolyl derivative **9** remained almost unchanged. The 3-, 4-dimethoxy substitution of compounds **10** and **11** increased the activity of the *N* 2-tolyl compound **11**. However, slight increases were also observed for the *N* 2-chlorophenyl derivative **10**. If the 3-methoxy functions in derivatives **6** and **7** were replaced with 3-benzyloxy functions in compounds **12** and **13** the activity of the *N* 2-chlorophenyl **12** compound remained almost unchanged and slight increases were observed for the *N* 2-tolyl derivative **13**. The 4-methoxy function in the aniline residue of compound **14** led to a reduced inhibitory activity and the combination with the 3-methoxy or the 4-methoxy function in derivatives **15** and **16** led to similar results. However, all three methoxy functions in compound **18** again increased the ABCB1 inhibitory activity. Therefore, it can be stated that methoxy functions placed in the 4-phenyl residue were favoured for the ABCB1 inhibitory activities which is in accordance with earlier publications that suggest such favourable methoxy functions act as hydrogen acceptor functions for a potential inhibitor binding to ABCB1 [15,16].

### 2.4. Antituberculostatic Drug Toxicity Enhancing Properties of 1,4-Dihydropyridines 

The observed ABCB1 inhibiting properties of our 1,4-dihydropyridines encouraged us to investigate a possible antituberculostatic drug toxicity enhancing effect of them in co-application with respective antituberculotic drugs by an inhibition of a mycobacterial efflux pump similar to ABCB1. Such drug toxicity enhancing effects may be a strategy to prevent drug resistance development of that drug. In the case of being a substrate of an efflux pump the intracellular level of a respective drug is reduced so that it becomes ineffective which means a drug resistance [17]. Furthermore, it has recently been demonstrated that such lowered drug levels induce the expression of respective efflux pumps in Mtb to enforce such a resistance development [18]. So far drug toxicity enhancing effects have only been described for a strongly limited number of used drugs which generally have their own pharmacological properties as antihypertensive or antidepressive drugs [19]. These properties do not allow their use as enhancers.

We investigated drug toxicity enhancing effects of the antituberculotic drug clofazimine. Clofazimine has originally been used as leprosy drug for the treatment of infections with *Mycobacterium leprae* [20]. Due to its potent activity against Mtb, clofazimine has been recommended by the World Health Organization in 2016 as a core second line agent against multidrug-resistant tuberculosis with favourable properties in tissue distribution, intracellular activity and prolonged half-life [1,21,22]. Thus, clofazimine became important for the design of second line regimens for the treatment of tuberculosis [1].

Clofazimine has been reported as an ABCB1 substrate in a cellular assay system of MDCK cells with the expression ABCB1 similar to our mouse T-lymphoma assay system although the cells have been different [23]. However, with the suggested ABCB1 substrate properties we expected a benefit of our compounds as inhibitors of ABCB1 in a co-application with clofazimine in Mtb cells to increase the clofazimine toxicity due to an inhibition of an antituberculotic efflux pump similar to ABCB1.

In our studies we used a concentration of clofazimine of 0.3 µg/mL to result in a mean reduction of the mycobacterial growth of 56%. Then we co-applicated clofazimine with each 1 µg/mL of selected compounds of our 1,4-dihydropyridines. We chose compounds **4**, **11** and **12**. All three compounds have not been practically active as antituberculotic drugs. Therefore, if an increased toxicity would result under co-application of those drugs with clofazimine the effect could be related to an inhibition of a mycobacterial efflux pump related to ABCB1.

For a co-application of compound **4** we found a further reduction of the bacterial growth to 28% which meant an increase of the growth inhibition of 44% compared to that of clofazimine alone (Table 2). Co-application of compound **11** resulted in a further reduction of growth to 23% which meant a growth inhibition increase of 55% compared to clofazimine. The use of compound **12** led to a mycobacterial growth reduction to 34% similar to compound **4**. Finally, we tested compound **13** which itself showed an antimycobacterial activity with a growth reduction of 19%. In combination with the clofazimine dose a total growth inhibition of 28% was observed which meant an increase of the inhibition of clofazimine by 54%.

A comparison of the increase of growth inhibition by the use of the four selected compounds with their ABCB1 inhibiting properties showed that the compounds with the lower ABCB1 affinities **4** and **12** resulted in the lower increase of growth inhibition. Compound **11** with highest ABCB1 affinities resulted in an enhanced growth inhibition activity. Compound **13** with a mean ABCB1 inhibiting activity compared to the other used compounds was found to cause an increase of growth inhibition similar to compound **11** due to its own activity in mycobacterial growth inhibition.

Therefore, our results suggested a compound activity on drug toxicity enhancing effects by an inhibition of a mycobacterial efflux pump similar to ABCB1 which is presently under discussion.

## 3. Material and Methods

### 3.1. Chemical Reagents and Instruments

Commercial reagents were used without further purification. The ^1^H-NMR spectra (500 MHz) were measured on an Inova Unity 500 (Varian) spectrometer using tetramethylsilane as internal standard. The ^1^H NMR spectra are shown in the Supplementary Materials. Thin layer chromatography (TLC) was performed on E. Merck 5554 silica gel plates. The high-resolution mass spectra were recorded on a Finnigan LCQ Classic mass spectrometer. Elemental analysis indicated by the symbols of the elements was within ±0.4% of the theoretical values and was performed using a Leco CHNS-932 apparatus.

### 3.2. General Procedure for the Synthesis of Compounds ***4***–***18***

Each 1 equivalent (4.2 mmol) of the aniline compound and of the aromatic aldehyde and 2 equivalents (8.2 mmol) of ethyl propiolate were heated in a small volume of ethanol under addition of acetic acid (10% *v*/*v*) under reflux conditions. The reaction proceeding was followed by TLC and mass spectrometry until no more starting compounds were detectable. Then acetic acid was removed from the solution by extraction with a brine solution (10% *m*/*v*) versus ethyl acetate three times. The unified ethyl acetate layer was dried over sodium sulphate and filtered. The filtrate was evaporated in vacuum and the remaining oil was purified by column chromatography using silica gel and an eluent mixture of cyclohexane and ethyl acetate (10%–20% *v*/*v*) if a direct isolation via crystallization failed. The compounds were finally recrystallized from mixtures of methanol and diethyl ether.

*Diethyl N-(2-chlorophenyl)-4-phenyl-1,4-dihydropyridine-3,5-dicarboxylate* (**4**). Yield 19%, white powder; mp 139–140 °C; ^1^H NMR (DMSO-*d*_6_) *δ* = 7.68–7.67 (m, 1H, 3- or 6-H of 2-Cl-Ph), 7.64 (m, 1H, 3- or 6-H of 2-Cl-Ph), 7.52–7.45 (m, 2H, 4-H, 5-H of 2-Cl-Ph), 7.36–7.34 (m, 2H, 2-, 6-H of Ph); 7.34 (s, 2H, 2-, 6-H), 7.29–7.26 (m, 2H, 3-, 5-H of Ph), 7.18–7.14 (m, 1H, 4-H of Ph), 4.79 (s, 1H, 4-H), 4.05–3.94 (m, 4H, COOC**H**_2_CH_3_), 1.11–1.08 (m, 6H, COOCH_2_C**H**_3_); ^13^C NMR (100 MHz, DMSO-*d*_6_) *δ* = 166.4 (CO), 144.4, 144.2, 139.3, 132.2, 130.7, 128.6, 127.7, 127.6, 125.7, 125.4, 123.8, 108.0 (aromat. C, 2-, 6-, 3-, 5-C), 60.9 (O**C**H_2_CH_3_), 37.4 (4-C), 14.2 (OCH_2_**C**H_3_); *m*/*z* (ESI) 412.55 (M + H^+^); anal. (C_23_H_22_ClNO_4_) calc. C 67.05, H 5.38, N 3.40; found C 66.75, H 5.18, N 3.22.

*Diethyl 4-phenyl-N-(o-tolyl)-1,4-dihydropyridine-3,5-dicarboxylate* (**5**). Yield 16%, white powder; mp 105–106 °C; ^1^H NMR (DMSO-*d*_6_) *δ* = 7.40–7.27 (m, 8H, 3-, 4-, 5-, 6-H of 2-CH_3_Ph and 2-, 3-, 5-, 6-H of Ph), 7.28 (s, 2H, 2-, 6-H), 7.18–7.14 (m, 1H, 4-H of Ph), 4.81 (s, 1H, 4-H), 4.05–3.93 (m, 4H, COOC**H**_2_CH_3_), 2.28 (s, 3H, 2-CH_3_Ph), 1.11–1.98 (m, 6H, COOCH_2_C**H**_3_); ^13^C NMR (100 MHz, DMSO-*d*_6_) *δ* = 166.8 (CO), 144.6, 144.0, 139.1, 131.3, 120.0, 128.6, 127.7, 126.5, 125.8, 123.7, 122.7, 109.0 (aromat. C, 2-, 6-, 3-, 5-C), 60.8 (O**C**H_2_CH_3_), 37.5 (4-C), 18.1 (CH_3_), 14.2 (OCH_2_**C**H_3_); *m*/*z* (ESI) 392.58 (M + H^+^); anal. (C_24_H_25_NO_4_) calc. C 73.64, H 6.44, N 3.58; found C 73.64, H 6.43, N 3.74.

*Diethyl N-(2-chlorophenyl)-4-(3-methoxyphenyl)-1,4-dihydropyridine-3,5-dicarboxylate* (**6**). Yield 20%, yellow-greenish powder; mp 127–128 °C; ^1^H NMR (DMSO-*d*_6_) δ = 7.68–7.66 (m, 1H, 3- or 6-H of 2-Cl-Ph), 7.63–7.61 (m, 1H, 3- or 6-H of 2-Cl-Ph), 7.51–7.45 (m, 2H, 4-, 5-H of 2-Cl-Ph), 7.34 (s, 2H, 2-, 6-H), 7.19 (t, *J* = 7.9 Hz, 1H, 5-H of 3-CH_3_OPh), 6.93 (dt, *J* = 7.9, 1.5 Hz, 1H, 6-H of 3-CH_3_OPh), 6.87 (dd, *J* = 2.6, 1.5 Hz, 1H, 2-H of 3-CH_3_OPh), 6.74 (ddd, *J* = 7.9, 2.6, 1.5 Hz, 1H, 4-H of 3-CH_3_OPh), 4.77 (s, 1H, 4-H), 4.07–3.95 (m, 4H, COOC**H**_2_CH_3_), 3.70 (s, 3H, 3-CH_3_O), 1.12–1.09 (m, 6H, COOCH_2_C**H**_3_); ^13^C NMR (100 MHz, DMSO-*d*_6_) *δ* = 166.5 (CO), 160.5, 144.6, 141.1, 138.7, 132.3, 130.3, 129.6, 127.3, 125.1, 123.4, 120.0, 113.1, 111.3, 107.3 (aromat. C, 2-, 6-, 3-, 5-C), 60.7 (O**C**H_2_CH_3_), 55.8 (OCH_3_), 37.5 (4-C), 14.2 (OCH_2_**C**H_3_; *m*/*z* (ESI) 442.51 (M + H^+^); anal. (C_24_H_24_ClNO_5_) calc. C 65.23, H 5.47, N 3.17; found C 65.11, H 5.55, N 2.90.

*Diethyl 4-(3-methoxyphenyl)-N-(o-tolyl)-1,4-dihydropyridine-3,5-dicarboxylate* (**7**). Yield 40%, white powder; mp 110–111 °C; ^1^H NMR (DMSO-*d*_6_) δ = 7.40–7.33 (m, 4H, 3-, 4-, 5-, 6-H of 2-CH_3_Ph), 7.28 (s, 2H, 2-, 6-H), 7.20 (t, *J* = 7.9 Hz, 1H, 5-H of 3-CH_3_OPh), 6.90 (dt, *J* = 7.9, 1.5 Hz, 1H, 6-H of 3-CH_3_OPh), 6.83 (dd, *J* = 2.6, 1.5 Hz, 1H, 2-H of 3-CH_3_OPh), 6.75 (ddd, *J* = 7.9, 2.6, 1.5 Hz, 1H, 4-H of 3-CH_3_OPh), 4.80 (s, 1H, 4-H), 4.06–3.95 (m, 4H, COOC**H**_2_CH_3_), 3.71 (s, 3H, 3-CH_3_OPh), 2.28 (s, 3H, 2-CH_3_Ph), 1.12–1.09 (m, 6H, COOCH_2_C**H**_3_); ^13^C NMR (100 MHz, DMSO-*d*_6_) *δ* = 166.6 (CO), 160.2, 143.9, 143.1, 138.1, 131.0, 129.6, 129.0, 126.3, 123.5, 122,4, 120.0, 113.1, 111.3, 108.0 (aromat. C, 2-, 6-, 3-, 5-C), 60.4 (O**C**H_2_CH_3_), 55.8 (OCH_3_), 37.8 (4-C), 14.1 (OCH_2_**C**H_3_); *m*/*z* (ESI) 422.28 (M + H^+^); anal. (C_25_H_27_NO_5_) calc. C 71.24, H 6.46, N 3.32; found C 71.27, H 6.46, N 3.49.

*Diethyl N-(2-chlorophenyl)-4-(4-methoxyphenyl)-1,4-dihydropyridine-3,5-dicarboxylate* (**8**). Yield 43%, yellow powder; mp 134–135 °C; ^1^H NMR (DMSO-*d*_6_) δ = 7.68–7.66 (m, 1H, 3- or 6-H of 2-Cl-Ph), 7.64–7.62 (m, 1H, 3- or 6-H of 2-Cl-Ph), 7.51–7.45 (m, 2H, 4-, 5-H of 2-Cl-Ph), 7.31 (s, 2H, 2-, 6-H), 7.26–7.23 (m, 2H, 2-, 6-H of 4-CH_3_OPh), 6.84–6.81 (m, 2H, 3-, 5-H of 4-CH_3_OPh), 4.72 (s, 1H, 4-H), 4.06–3.94 (m, 4H, COOC**H**_2_CH_3_), 3.69 (s, 3H, 4-CH_3_O), 1.12–1.09 (m, 6H, COOCH_2_C**H**_3_); ^13^C NMR (100 MHz, DMSO-*d*_6_) *δ* = 166.8 (CO), 157.6, 144.1, 138.7, 136.7, 132.1, 130.2, 130.0, 127.2, 125.2, 123.4, 114.2, 107.4 (aromat. C, 2-, 6-, 3-, 5-C), 60.3 (O**C**H_2_CH_3_), 55.4 (OCH_3_), 37.3 (4-C), 14.2 (OCH_2_**C**H_3_); *m*/*z* (ESI) 442.54 (M + H^+^); anal. (C_24_H_24_ClNO_5_) calc. C 65.23, H 5.47, N 3.17; found C 65.32, H 5.46, N 3.17.

*Diethyl 4-(4-methoxyphenyl)-N-(o-tolyl)-1,4-dihydropyridine-3,5-dicarboxylate* (**9**). Yield 21%, yellow powder; mp 87–89 °C; ^1^H NMR (DMSO-*d*_6_) δ = 7.40–7.31 (m, 4H, 3-, 4-, 5-, 6-H of 2-CH_3_Ph), 7.25 (s, 2H, 2-, 6-H), 7.23–7.20 (m, 2H, 2-, 6-H of 4-CH_3_OPh), 6.85–6.82 (m, 2H, 3-, 5-H of 4-CH_3_OPh), 4.75 (s, 1H, 4-H), 4.05–3.93 (m, 4H, COOC**H**_2_CH_3_), 3.70 (s, 3H, 4-CH_3_OPh), 2.28 (s, 3H, 2-CH_3_Ph), 1.12–1.09 (m, 6H, COOCH_2_C**H**_3_); ^13^C NMR (100 MHz, DMSO-*d*_6_) *δ* = 166.6 (CO), 157.2, 144.3, 138.4, 136.8, 131.6, 130.4, 126.9, 123.6, 122.7, 114.1, 108.2 (aromat. C, 2-, 6-, 3-, 5-C), 60.7 (O**C**H_2_CH_3_), 55.4 (OCH_3_), 37.8 (4-C), 18.0 (CH_3_), 14.1 (OCH_2_**C**H_3_); *m*/*z* (ESI) 422.88 (M + H^+^); anal. (C_25_H_27_NO_5_) calc. C 71.24, H 6.46, N 3.32; found C 70.95, H 6.41, N 3.31. 

*Diethyl N-(2-chlorophenyl)-4-(3,4-dimethoxyphenyl)-1,4-dihydropyridine-3,5-dicarboxylate* (**10**). Yield 20%, white powder; mp 106–108 °C; ^1^H NMR (DMSO-*d*_6_) δ = 7.68–7.66 (m, 1H, 3- or 6-H of 2-Cl-Ph), 7.63–7.61 (m, 1H, 3- or 6-H of 2-Cl-Ph), 7.51–7.45 (m, 2H, 4-, 5-H of 2-Cl-Ph), 7.32 (s, 2H, 2-, 6-H), 6.88–6.85 (m, 3H, 2-, 5-, 6-H of 3,4-(CH_3_O)_2_Ph), 4.73 (s, 1H, 4-H), 4.07–3.96 (m, 4H, COOC**H**_2_CH_3_), 3.70, 3.69 (2 × s, 6H, 3,4-(CH_3_O)_2_), 1.13–1.10 (m, 6H, COOCH_2_C**H**_3_); ^13^C NMR (100 MHz, DMSO-*d*_6_) *δ* = 166.4 (CO), 149.7, 146.8, 144.1, 138.7, 135.5, 132.2, 130.7, 127.6, 125.4, 123.8, 122.3, 114.1, 112.3, 108.1 (aromat. C, 2-, 6-, 3-, 5-C), 60.4 (O**C**H_2_CH_3_), 55.9, 55.8 (OCH_3_), 37.8 (4-C), 18.0 (CH_3_), 14.1 (OCH_2_**C**H_3_); *m*/*z* (ESI) 472.88 (M + H^+^); anal. (C_25_H_26_ClNO_6_) calc. C 63.63, H 5.55, N 2.97; found C 63.53, H 5.49, N 2.91. 

*Diethyl 4-(3,4-dimethoxyphenyl)-N-(o-tolyl)-1,4-dihydropyridine-3,5-dicarboxylate* (**11**). Yield 35%, yellow powder; mp 110–111 °C; ^1^H NMR (DMSO-*d*_6_) δ = 7.41–7.34 (m, 4H, 3-, 4-, 5-, 6-H of 2-CH_3_Ph), 7.26 (s, 2H, 2-, 6-H), 6.88–6.81 (m, 3H, 2-, 5-, 6-H of 3,4-(CH_3_O)_2_Ph), 4.76 (s, 1H, 4-H), 4.07–3.95 (m, 4H, COOC**H**_2_CH_3_), 3.71, 3.69 (2 × s, 6H, 3,4-(CH_3_O)_2_), 2.29 (s, 3 H, 2-CH_3_-Ph), 1.14–1.10 (m, 6H, COOCH_2_C**H**_3_); ^13^C NMR (100 MHz, DMSO-*d*_6_) *δ* = 166.4 (CO), 149.3, 146.9, 144.3, 138.2, 135.83, 131.4, 129.8, 126.1, 123.4, 122.4, 122.0, 113.9, 112.1, 107.8 (aromat. C, 2-, 6-, 3-, 5-C), 60.6 (O**C**H_2_CH_3_), 55.9, 56.1 (OCH_3_), 37.9 (4-C), 18.3 (CH_3_), 14.2 (OCH_2_**C**H_3_); *m*/*z* (ESI) 453.05 (M + H^+^); anal. (C_26_H_29_NO_6_) calc. C 69.16, H 6.47, N 3.10; found C 68.88, H 6.42, N 3.26. 

*Diethyl 4-(3-(benzyloxy)phenyl)-N-(2-chlorophenyl)-1,4-dihydropyridine-3,5-dicarboxylate* (**12**). Yield 10%, yellow powder; mp 106–110 °C; ^1^H NMR (DMSO-*d*_6_) δ = 7.68–7.66 (m, 1H, 3- or 6-H of 2-Cl-Ph), 7.63–7.62 (m, 1H, 3- or 6-H of 2-Cl-Ph), 7.51–7.45 (m, 2H, 4-, 5-H of 2-Cl-Ph), 7.42–7.40 (m, 2H, 2-, 6-H of BnO), 7.38–7.34 (m, 2H, 3-, 5-H of BnO), 7.34 (s, 2H, 2-, 6-H), 7.32–7.29 (m, 1H, 4-H of BnO), 7.19 (t, *J* = 7.8 Hz, 1H, 5-H of 3-BnOPh), 6.96–8.94 (m, 2H, 2-, 6-H of 3-BnOPh), 6.83 (ddd, *J* = 7.8, 2.5, 1.1 Hz, 4-H of 3-BnOPh), 5.03 (s, 2H, Ph-O-C**H**_2_-Ph), 4.77 (s, 1H, 4-H), 4.06–3.95 (m, 4H, COOC**H**_2_CH_3_), 1.12–1.10 (m, 6H, COOCH_2_C**H**_3_); ^13^C NMR (100 MHz, DMSO-*d*_6_) *δ* = 166.8 (CO), 160.5, 143.9, 143.8, 138.4, 136.7, 132.1, 130.7, 129.6, 128.9, 127.7, 127.6, 127.1, 125.4, 123.8, 120.0, 113.1, 111.3, 107.8 (aromat. C, 2-, 6-, 3-, 5-C), 70.8 (OCH_2_), 60.9 (O**C**H_2_CH_3_), 55.9, 56.1 (OCH_3_), 38.1 (4-C), 14.2 (OCH_2_**C**H_3_); *m*/*z* (ESI) 519.32 (M + H^+^); anal. (C_30_H_28_ClNO_5_) calc. C 69.56, H 5.45, N 2.70; found C 69.60, H 5.43, N 2.66. 

*Diethyl 4-(3-(benzyloxy)yphenyl)-N-(o-tolyl)-1,4-dihydropyridine-3,5-dicarboxylate* (**13**). Yield 49%, yellow powder; mp 117–118 °C; ^1^H NMR (DMSO-*d*_6_) δ = 7.43–7.28 (m, 9H, aromatic H of *N*-tolyl and OBn), 7.28 (s, 2H, 2-, 6-H), 7.20 (t, *J* = 8.1 Hz, 1H, 5-H of 3-BnO-Ph), 6.92–6.91 (m, 2H, 2-, 6-H of 3-BnO-Ph), 6.83 (ddd, *J* = 8.1, 2.4, 1.0 Hz, 1H, 4-H of 3-BnOPh), 5.04 (s, 2H, Ph-O-C**H**_2_-Ph), 4.80 (s, 1H, 4-H), 4.06–3.94 (m, 4H, COOC**H**_2_CH_3_), 2.27 (s, 3H, 2-CH_3_Ph), 1.12–1.09 (m, 6H, COOCH_2_C**H**_3_); ^13^C NMR (100 MHz, DMSO-*d*_6_) *δ* = 166.7 (CO), 160.9, 144.2, 142.9, 138.3, 136.4, 130.9, 129.8, 129.3, 128.5, 127.3, 127.0, 126.1, 123.4, 122.6, 120.2, 112.8, 108.2 (aromat. C, 2-, 6-, 3-, 5-C), 71.2 (OCH_2_), 60.5 (O**C**H_2_CH_3_), 37.6 (4-C), 18.0 (CH_3_), 14.2 (OCH_2_**C**H_3_); *m*/*z* (ESI) 497.71 (M + H^+^); anal. (C_31_H_31_NO_5_) calc. C 74.83, H 6.28, N 2.81; found C 74.86, H 6.36, N 2.90. 

*Diethyl N-(4-methoxyphenyl)-4-phenyl-1,4-dihydropyridine-3,5-dicarboxylate* (**14**). Yield 63%, yellow powder; mp 116–117 °C; ^1^H NMR (DMSO-*d*_6_) *δ* = 7.50 (s, 2H, 2-, 6-H), 7.42–7.38 (m, 2H, 3-, 5-H of 4-CH_3_O-Ph), 7.27–7.23 (m, 4H, 2-, 3-, 5-, 6-H of 4-Ph), 7.16–7.12 (m, 1H, 4-H of 4-Ph), 7.04–7.00 (m, 2H, 2-, 6-H of 4-MeO-Ph), 4.78 (s, 1H, 4-H), 4.07–3.93 (m, 4H, COOC**H**_2_CH_3_), 3.77 (s, 3H, 4-MeO), 1.12–1.09 (m, 6H, COOCH_2_C**H**_3_); ^13^C NMR (100 MHz, DMSO-*d*_6_) *δ* = 167.2 (CO), 153.3, 144.4, 139.3, 133.5, 128.5, 127.7, 125.7, 123.5, 115.1, 108.5 (aromat. C, 2-, 6-, 3-, 5-C), 61.3 (O**C**H_2_CH_3_), 55.8 (OCH_3_), 38.3 (4-C), 14.3 (OCH_2_*C*H_3_); *m*/*z* (ESI) 408.64 (M + H^+^); anal. (C_24_H_25_NO_5_) calc. C 70.75, H 6.18, N 3.44; found C 70.39, H 6.27, N 3.39.

*Diethyl 4-(3-methoxyphenyl)-N-(4-methoxyphenyl)-1,4-dihydropyridine-3,5-dicarboxylate* (**15**). Yield 53%, yellow powder; mp 120–121 °C; ^1^H NMR (DMSO-*d*_6_) *δ* = 7.50 (s, 2H, 2-, 6-H), 7.41–7.38 (m, 2H, 3-, 5-H of 4-CH_3_O-Ph), 7.18 (t, *J* = 7.9 Hz, 1H, 5-H of 3-CH_3_O-Ph), 7.04–7.01 (m, 2H, 2-, 6-H of 4-CH_3_O-Ph), 6.84 (dt, *J* = 7.9, 1.2 Hz, 1H, 6-H of 3-CH_3_O-Ph), 6.77 (dd, *J* = 2.6, 1.2 Hz, 1H, 2-H of 3-CH_3_O-Ph), 6.73 (ddd, *J* = 7.9, 2.6, 1.7 Hz, 1H, 4-H of 3-CH_3_O-Ph), 4.76 (s, 1H, 4-H), 4.08–3.96 (m, 4H, COOC**H**_2_CH_3_), 3.77 (s, 3H, 4-CH_3_O), 3.69 (s, 3H, 3-CH_3_O), 1.14–1.11 (m, 6H, COOCH_2_C**H**_3_); ^13^C NMR (100 MHz, DMSO-*d*_6_) *δ* = 167.2 (CO), 160.5, 153.2, 143.2, 138.9, 133.5, 129.6, 123.3, 120.0, 115.3, 113.1, 111.3, 108.1 (aromat. C, 2-, 6-, 3-, 5-C), 61.7 (O**C**H_2_CH_3_), 55.8 (OCH_3_), 38.2 (4-C), 14.2 (OCH_2_*C*H_3_); *m*/*z* (ESI) 438.44 (M + H^+^); anal. (C_25_H_27_NO_6_) calc. C 68.64, H 6.22, N 3.20; found C 68.14, H 6.17, N 3.17.

*Diethyl 1,4-bis(4-methoxyphenyl)-1,4-dihydropyridine-3,5-dicarboxylate* (**16**). Yield 56%, yellow powder; mp 120–121 °C; ^1^H NMR (DMSO-*d*_6_) *δ* = 7.48 (s, 2H, 2-, 6-H), 7.41–7.38 (m, 2H, 3-, 5-H of 4- CH_3_O-*N*-Ph), 7.17-7.14 (m, 2H, 2-, 6-H of 4-CH_3_O-4-Ph), 7.04–7.01 (m, 2H, 2-, 6-H of 4-CH_3_O-*N*-Ph), 6.82–6.79 (m, 2H, 3-, 5-H of 4-CH_3_O-4-Ph), 4.71 (s, 1H, 4-H), 4.07–3.95 (m, 4H, COOC**H**_2_CH_3_), 3.77 (s, 3H, 4-CH_3_O-*N*-Ph), 3.68 (s, 3H, 4-CH_3_O-4-Ph), 1.13–1.11 (m, 6H, COOCH_2_C**H**_3_); ^13^C NMR (100 MHz, DMSO-*d*_6_) *δ* = 167.0 (CO), 157.6, 153.3, 136.7, 139.4, 133.2, 130.0, 123.5, 115.1, 114.2, 108.1 (aromat. C, 2-, 6-, 3-, 5-C), 61.4 (O**C**H_2_CH_3_), 55.8 (OCH_3_), 38.3 (4-C), 14.1 (OCH_2_*C*H_3_); *m*/*z* (ESI) 438.15 (M + H^+^); anal. (C_25_H_27_NO_6_) calc. C 68.64, H 6.22, N 3.20; found C 68.35, H 6.04, N 3.13.

*Diethyl 4-(3,4-methoxyphenyl)-N-(4-methoxyphenyl)-1,4-dihydropyridine-3,5-dicarboxylate* (**17**). Yield 51%, yellow powder; mp 138–139 °C; ^1^H NMR (DMSO-*d*_6_) *δ* = 7.49 (s, 2H, 2-, 6-H), 7.41–7.38 (m, 2H, 3-, 5-H of 4-CH_3_O-*N*-Ph), 7.04–7.01 (m, 2H, 2-, 6-H of 4-CH_3_O-*N*-Ph), 6.83 (d, *J* = 1H, 5-H of 3,4-CH_3_O-Ph), 6.81 (d, *J* = 2.1 Hz, 1H, 2-H of 3,4-CH_3_O-Ph), 6.75 (dd, *J* = 8.3, 2.1 Hz, 1H, 6-H of 3,4-CH_3_O-Ph), 4.71 (s, 1H, 4-H), 4.08–3.97 (m, 4H, COOC**H**_2_CH_3_), 3.77 (s, 3H, 4-CH_3_O-*N*-Ph), 3.68 (s, 6H, 3,4-CH_3_O-4-Ph), 1.15–1.12 (m, 6H, COOCH_2_C**H**_3_); ^13^C NMR (100 MHz, DMSO-*d*_6_) *δ* = 167.1 (CO), 153.4, 149.7, 146.8, 138.5, 135.5, 133.5, 123.5, 122.3, 115.3, 114.1, 112.3, 108.3 (aromat. C, 2-, 6-, 3-, 5-C), 61.4 (O**C**H_2_CH_3_), 55.8 (OCH_3_), 38.5 (4-C), 14.2 (OCH_2_**C**H_3_); *m*/*z* (ESI) 468.52 (M + H^+^); anal. (C_26_H_29_NO_7_) calc. C 66.80, H 6.25, N 3.00; found C 66.40, H 6.14, N 2.75.

*Diethyl 4-(3-(benzyloxy)yphenyl)-N-(4-methoxyphenyl)-1,4-dihydropyridine-3,5-dicarboxylate* (**18**). Yield 55%, yellow powder; mp 127–128 °C; ^1^H NMR (DMSO-*d*_6_) δ = 7.49 (s, 2H, 2-, 6-H), 7.41–7.37 (m, 4H, 3-, 5-H of 4-CH_3_O-Ph and 2-, 6-H of OBn), 7.36–7.32 (m, 2H, 3-, 5-H of OBn), 7.31–7.27 (m, 1H, 4-H of OBn), 7.19–7.16 (m, 1H, 5-H of 3-BnOPh), 7.04–7.01 (m, 2H, 2-, 6-H of 4-CH_3_O-Ph), 6.87–6.80 (m, 3H, 2-, 4-, 6-H of 3-BnOPh), 5.03 (s, 2H, Ph-O-CH_2_-Ph), 4.76 (s, 1H, 4-H), 4.07–3.96 (m, 4H, COOC**H**_2_CH_3_), 3.77 (s, 3H, CH_3_O), 1.13–1.11 (m, 6H, COOCH_2_C**H**_3_); ^13^C NMR (100 MHz, DMSO-*d*_6_) *δ* = 167.2 (CO), 160.5, 153.1, 143.2, 139.3, 136.7, 133.5, 129.6, 128.9, 127.6, 127.0, 123.3, 120.0, 115.1, 113.1, 111.2, 107.9 (aromat. C, 2-, 6-, 3-, 5-C), 70.6 (OCH_2_Ph), 61.7 (O**C**H_2_CH_3_), 55.8 (OCH_3_), 38.5 (4-C), 14.2 (OCH_2_*C*H_3_); *m*/*z* (ESI) 514.51 (M + H^+^); anal. (C_31_H_31_NO_6_) calc. C 72.50, H 6.08, N 2.73; found C 72.32, H 6.08, N 2.65. 

### 3.3. ABCB1 Inhibitory Activity

Two cell lines, a mouse T-lymphoma parental cell line L5178Y and an ABCB1 expressing subline L5178Y *mdr*, which resulted from retrovirus-mediated gene transfection, were cultured in McCoy’s 5A medium which was supplemented with 10% calf serum and l-glutamine (2 mM) at 37 °C under a carbon dioxide atmosphere (5%). The ABCB1 expressing subline was cultured under addition of colchicine in a concentration of 60 ng/mL to ensure a survival of only ABCB1-overexpressing cells. The cell suspensions were diluted three times a week with fresh medium in a relation of 1 to 20.

Both cell lines were adjusted to a number of one million cells per mL. After centrifugation at 2000 rpm the upper layer was removed and the remaining cells were resuspended in medium. Each 0.5 mL of the cell suspensions were placed in an Eppendorf tube and supplemented with the inhibitor from stock solutions to reach a final concentration of 1 µM for testing. Incubation followed for 20 min. Then 0.5 µL of a rhodamine 123 stock solution (0.5 mM) was added and incubation was continued for an additional 40 min. Then centrifugation followed again at 2000 rpm. The medium was removed and the cell suspension was washed with phosphate-buffered saline (PBS)buffer twice at a pH of 7.4. Then the fluorescence was measured in both cell lines and the fluorescence values were each related to the fluorescence of the untreated control to give the final FAR value by relating both corrected fluorescence values of the ABCB1-overexpressing cell line and the parental cell line. The determination of the FAR value followed the equation:

MDR treated/MDR untreated control

*FAR* = Parental treated/Parental untreated control

### 3.4. Mtb Growth Inhibition Assay 

For growth analyses green fluorescence-expressing Mtb H37Rv bacteria were used as described [24]. In short details 2 × 10^6^ bacteria were cultured in 7H9 medium supplemented with 10% oleic acid–albumin–dextrose–catalase (OADC), 0.05% Tween 80 and 0.2% glycerol in a total volume of 100 µL in a black 96-well plate with a clear bottom (Corning Inc.) sealed with an air-permeable membrane (Porvair Sciences). Direct bacterial growth was measured under compound concentrations of 1 µg/mL similar to earlier cited studies where higher concentrations have been used [7]. IC_50_ values could not be determined due to occurring problems with the compound solubility at the higher concentrations. In the drug-toxicity enhancing studies a clofazimine concentration of 0.3 µg/mL and a combination of clofazimine (0.3 µg/mL) and the respective 1,4-dihydropyridine (1 µg/mL) were used. The bacterial growth was measured in triplicates as relative light units at 528 nm after excitation at 485 nm in a fluorescence microplate reader after seven days of culture (Synergy 2, Biotek). 

## 4. Conclusions

The number of drugs used in antituberculotic therapies is strongly limited. Moreover, drugs used in case of an MDR treatment of Mtb suffer from low efficacy, part toxicity and a long duration with a limited successful outcome. Therefore, there is a need for novel antituberculotic drugs and also drugs that may enhance the activity of drugs currently in use. This is a challenge because presently drugs that have been investigated cannot be used in therapy because of their own pharmacological effects. We identified nonclassical 1,4-dihydropyridines as novel dually acting compounds with antituberculotic activities and drug toxicity enhancing activities. Favourable substitution patterns of the 1,4-dihydropyridine scaffold have been 2-toly substituents at the nitrogen phenyl substituent and a methoxy function placed in both aryl substituents. However, for a more detailed structure–activity discussion the target structure has to be identified in future studies. In case of antibacterial drug development, the target structures are mostly unclear in the early state as in the case of the recently discovered lipolanthines [25]. The determined ABCB1 inhibitory activities promised effects on a mycobacterial efflux pump similar to ABCB1. Therefore, compounds with varying ABCB1 activities were evaluated to enhance the antituberculotic activities of clofazimine as ABCB1 substrate that may be transported by a mycobacterial efflux pump related to ABCB1. The best results as enhancers were observed for those compounds with the best ABCB1 affinities and with combined antituberculotic and ABCB1 inhibiting properties. Perspective lead compounds have been identified which prove the principle of an effective dual activity to combat Mtb and Mtb drug resistance. 

## Figures and Tables

**Table 1:** *Mycobacterium tuberculosis* (Mtb) cell growth inhibition and ABCB1-inhibiting properties of target compounds **4**–**18** expressed as fluorescence activity ratio (FAR) values.

Cpd.	R^1^	R^2^	Growth Inhibition (%) ^(a)^	FAR Value ^(a)^
**4**	2-Cl	H	9	11.20
**5**	2-Me	H	34	8.19
**6**	2-Cl	3-OMe	37	32.62
**7**	2-Me	3-OMe	40	33.59
**8**	2-Cl	4-OMe	24	52.49
**9**	2-Me	4-OMe	2	30.80
**10**	2-Cl	3-, 4-OMe	9	59.76
**11**	2-Me	3-, 4-OMe	n.a. ^(b)^	68.30
**12**	2-Cl	3-OBn	n.a. ^(b)^	31.46
**13**	2-Me	3-OBn	17	42.29
**14**	4-OMe	H	60	3.9
**15**	4-OMe	3-OMe	41	3.4
**16**	4-OMe	4-OMe	35	1.2
**17**	4-OMe	3-, 4-OMe	59	5.2
**18**	4-OMe	3-OBn	7	n.d. ^(c)^
INH			97	n.d. ^(c)^
Verapamil			n.d. ^(c)^	4.10
Tariquidar			n.d. ^(c)^	15.3

^(a)^ Mean of three determinations; ^(b)^ not active; ^(c)^ not determined.

**Table 2:** Mtb cell effects of selected target compounds **4** and **11**–**13** under clofazimine co-application.

	Growth (%)	Growth (%) ^(a)^	Percentual Growth
Cpd.	Target Compound Only	Clofazimine + Target Compound Inhibition Increase (%)	
**4**	96	28	44
**11**	100	23	55
**12**	100	34	44
**13**	81	28	54

^(a)^ Mean of three determinations.

## Supplementary Materials

The supplementary materials are available online, Spectra S1: ^1^H NMR spectra.
