# Supplementary material for: Dually Acting Nonclassical 1,4-Dihydropyridines Promote the Anti-Tuberculosis (Tb) Activities of Clofazimine
Source: Molecules. 2019 Aug 8;24(16):2873. doi: 10.3390/molecules24162873 (PMC6720424; doi:10.3390/molecules24162873)
Supplement: Supplementary file 1 [file molecules-24-02873-s001.pdf]

Supplementary Materials

Dually acting nonclassical 1,4-dihydropyridines promote the anti-Tuberculosis (Tb) activities of clofazimine

Fabian Lentz [1], Norbert Reiling [2], Gabriella Spengler [3], Annamária Kincses[3], Andrea Csonka[3], Joseph Molnár [3] and Andreas Hilgeroth [1,*]

[1] Institute of Pharmacy, Martin-Luther-University Halle-Wittenberg, 06120 Halle, Germany; andreas.hilgeroth@pharmazie.uni-halle.de
[2] Research Center of Borstel, Leibniz Lung Center, 23845 Borstel, Germany; nreiling@fz-borstel.de
[3] Department of Medical Microbiology, University of Szeged, 6720 Szeged, Hungary; molnar.jozsef@med.u-szeged.hu
* Correspondence: andreas.hilgeroth@pharmazie.uni-halle.de; Tel.: +49-345-55-25168

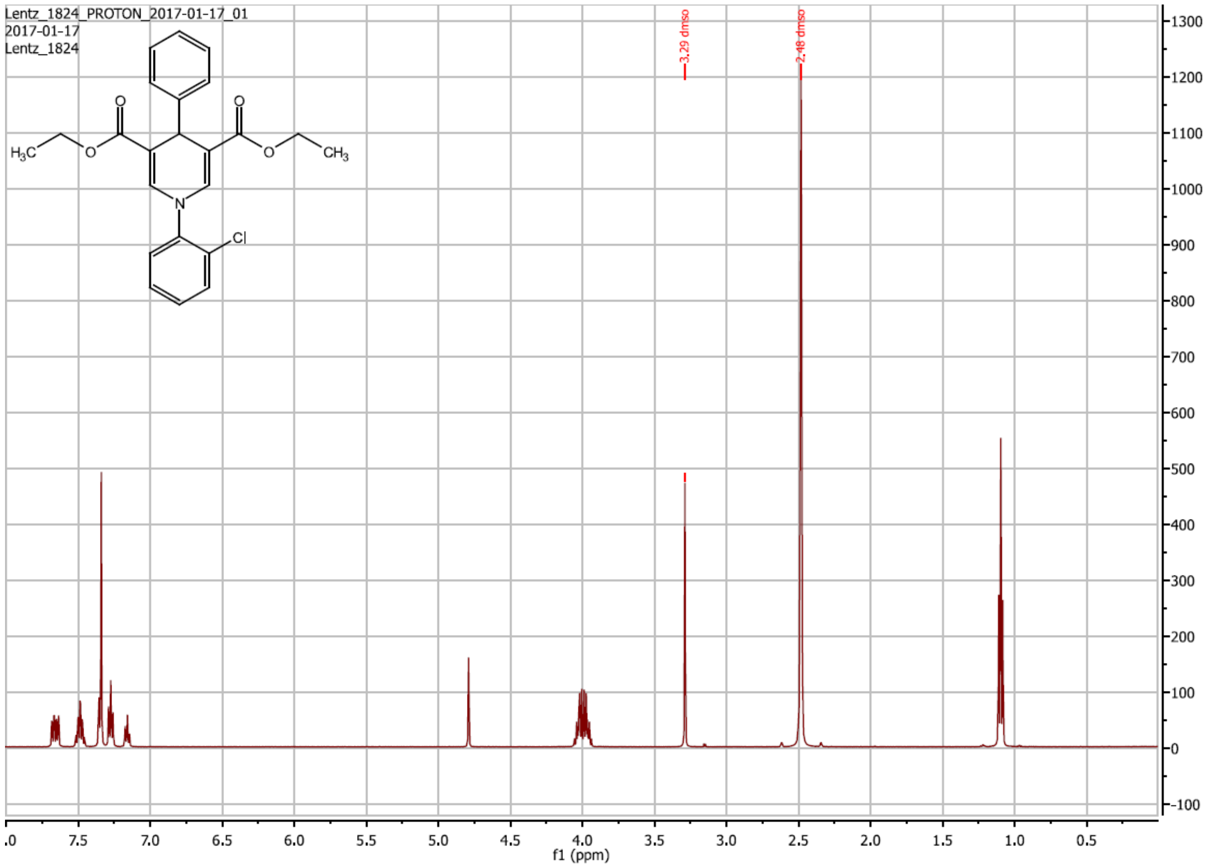

^{1}H NMR spectrum of compound **4**

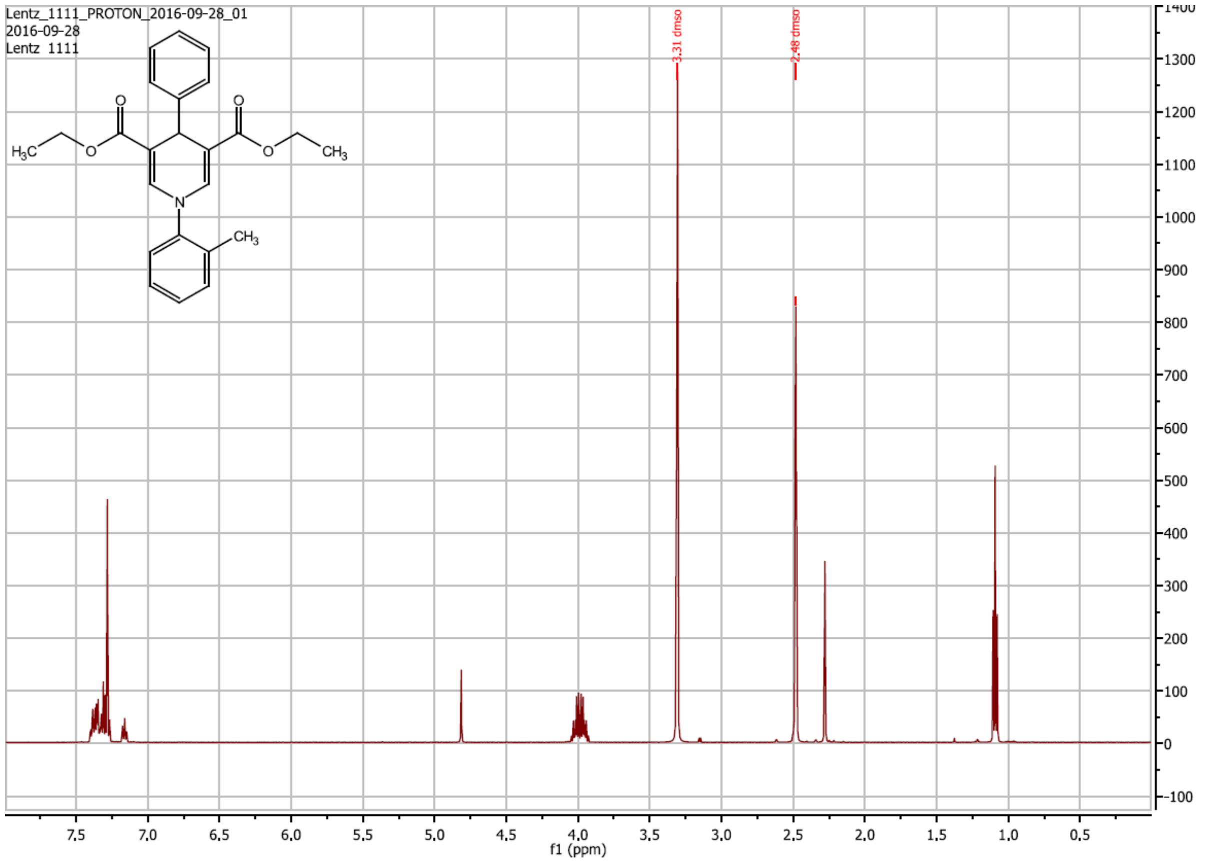

^{1}H NMR spectrum of compound **5**

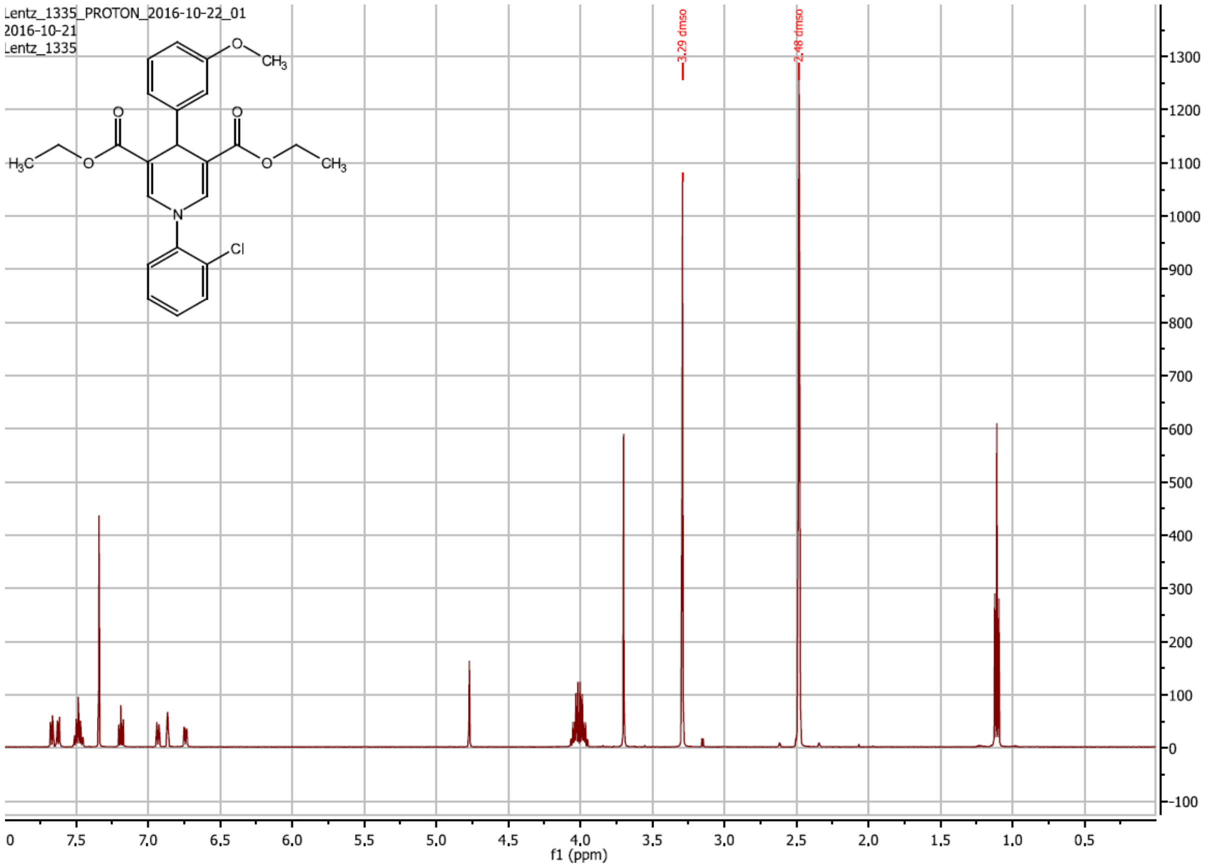

^{1}H NMR spectrum of compound **6**

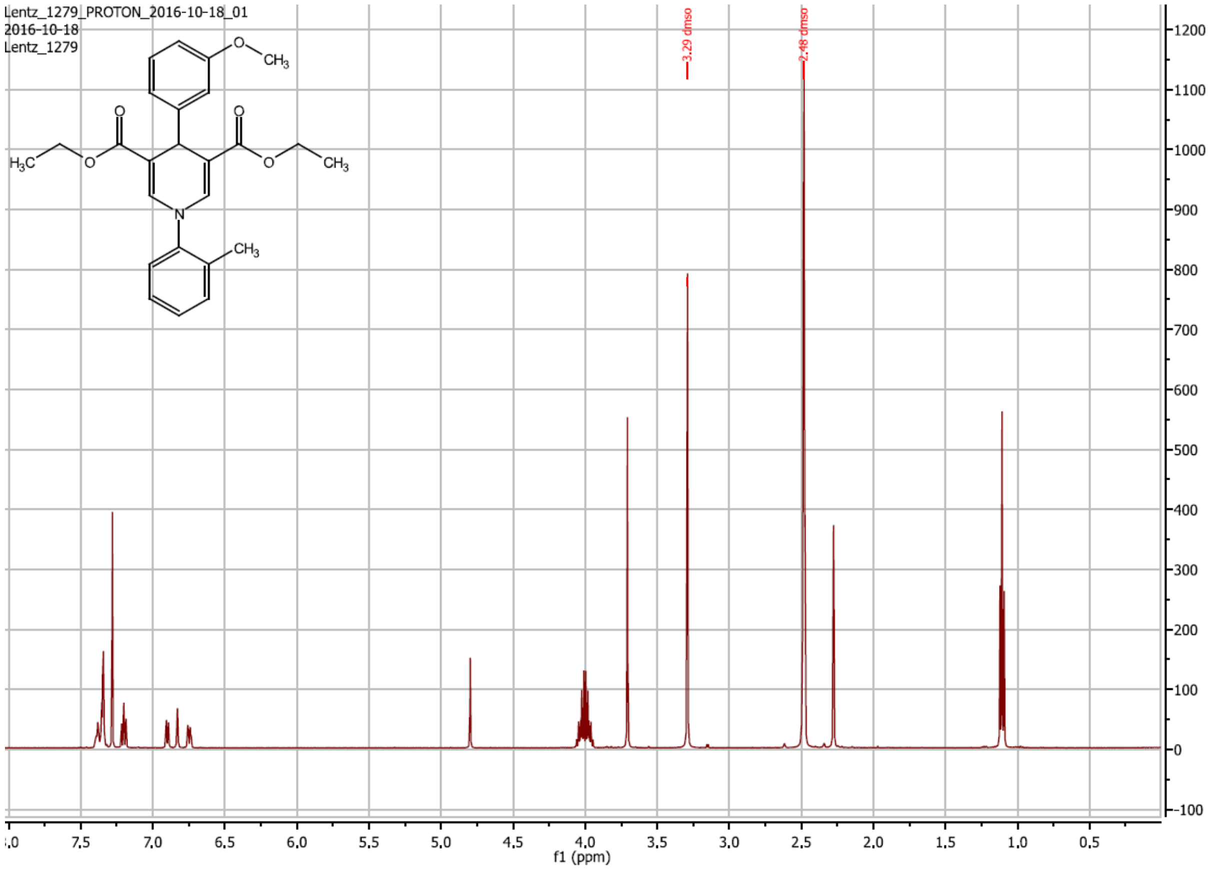

^{1}H NMR spectrum of compound **7**

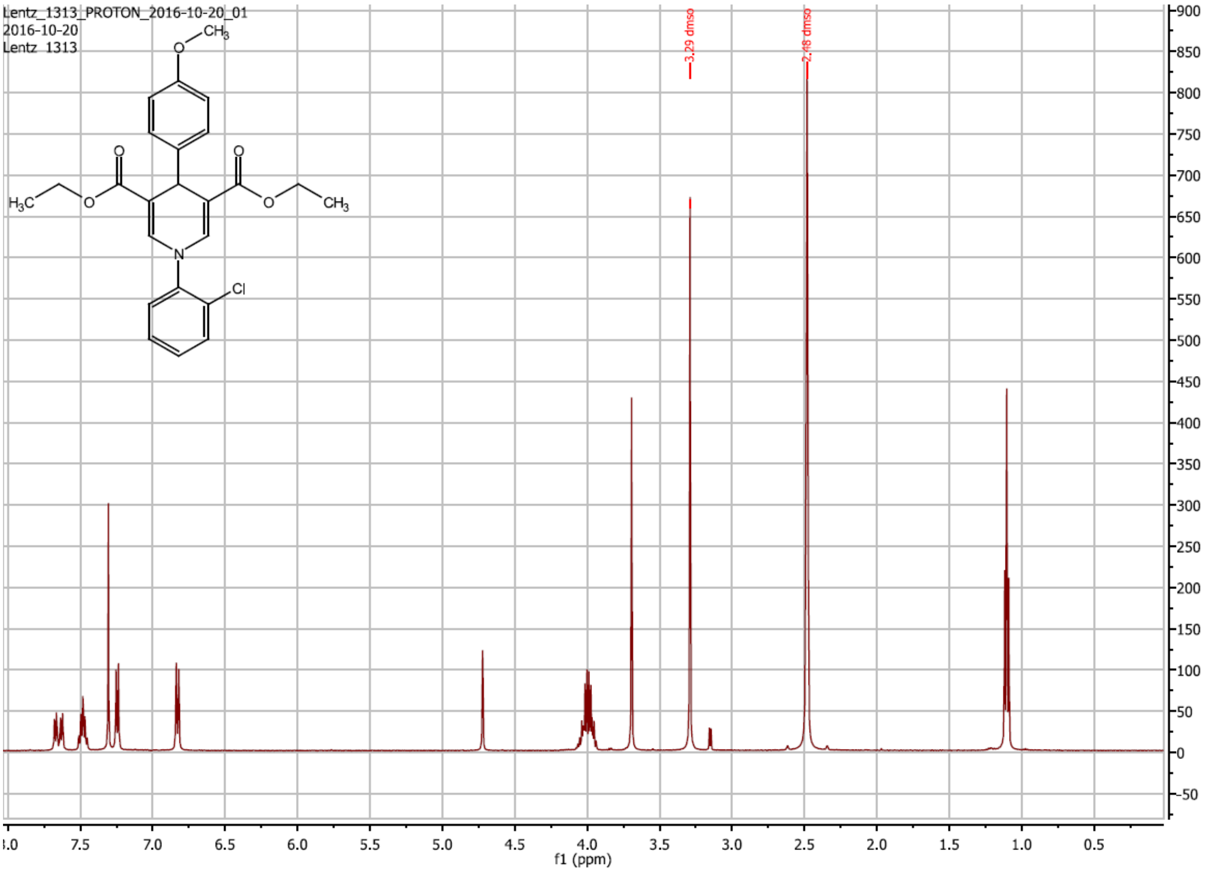

^{1}H NMR spectrum of compound **8**

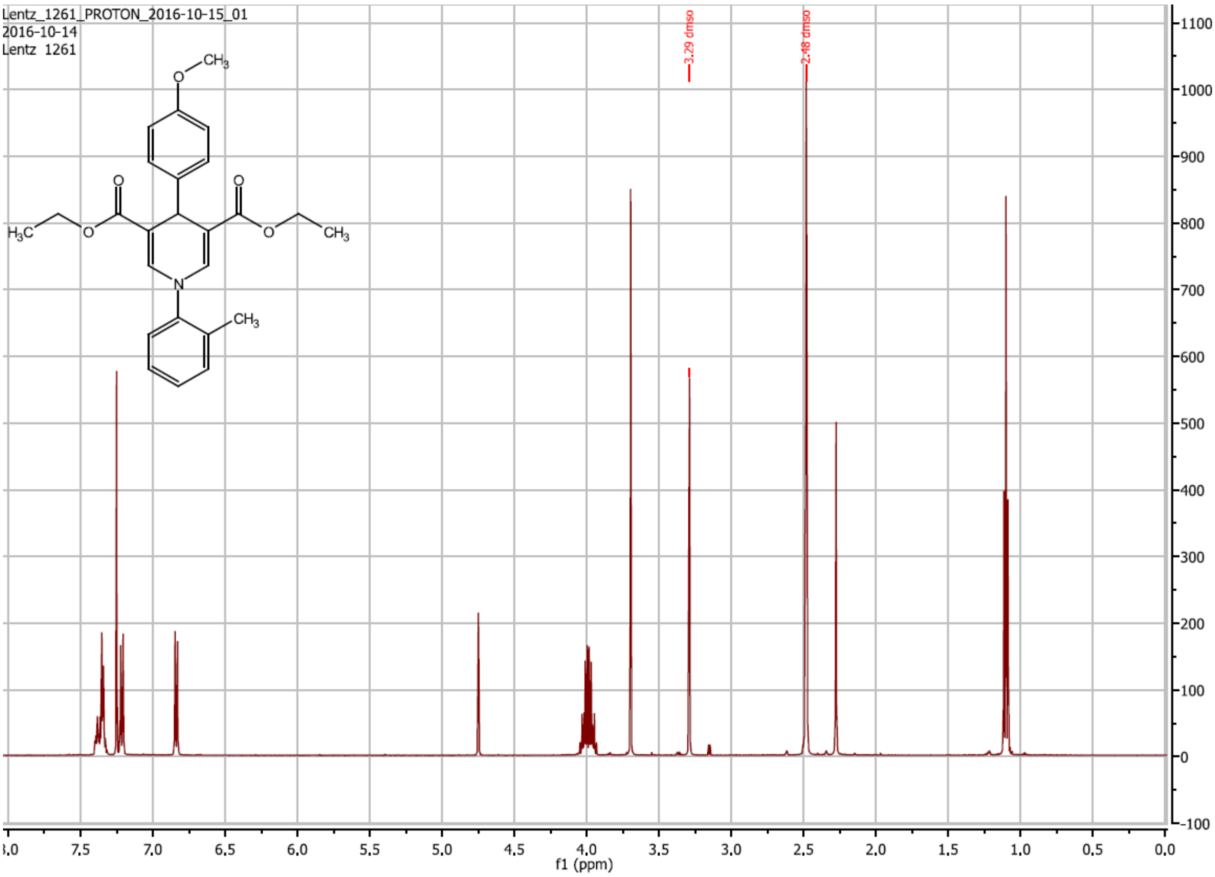

^{1}H NMR spectrum of compound **9**

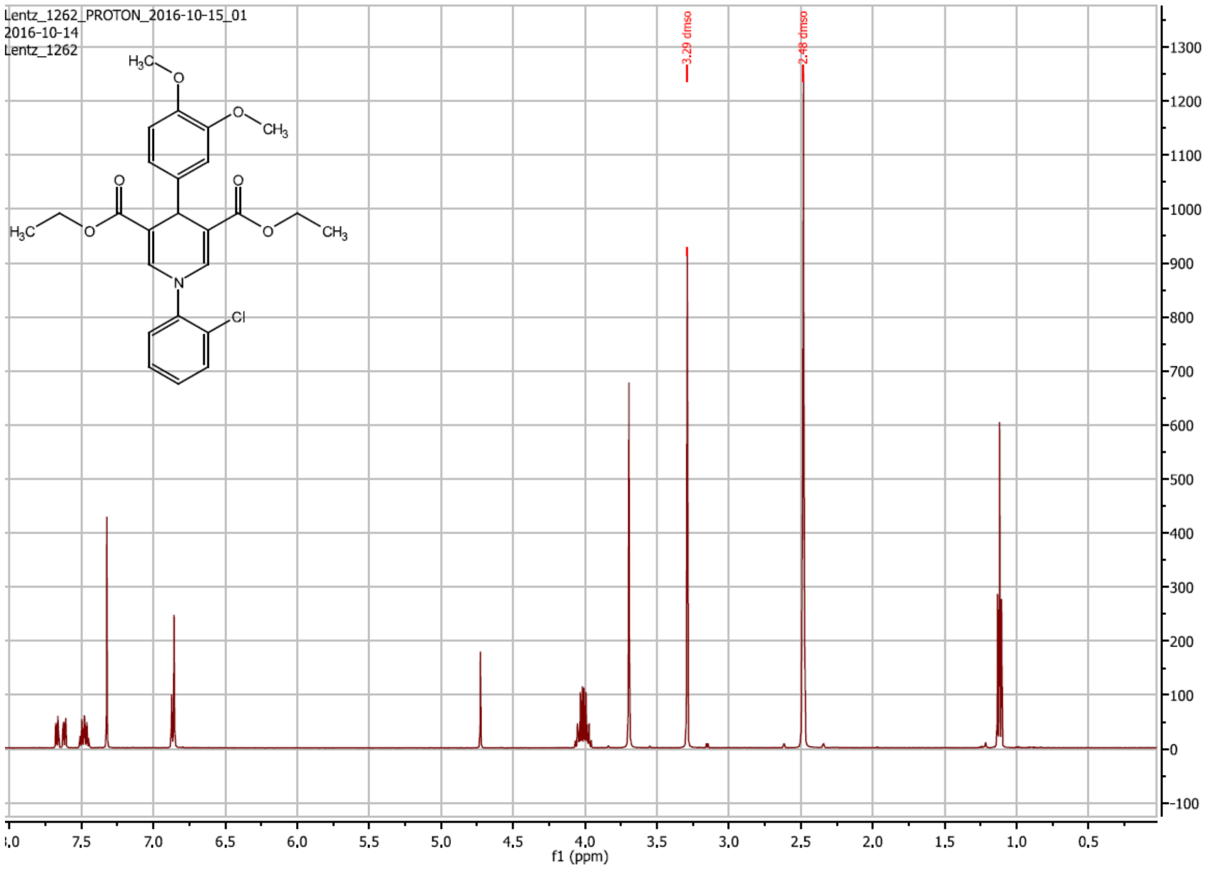

^{1}H NMR spectrum of compound **10**

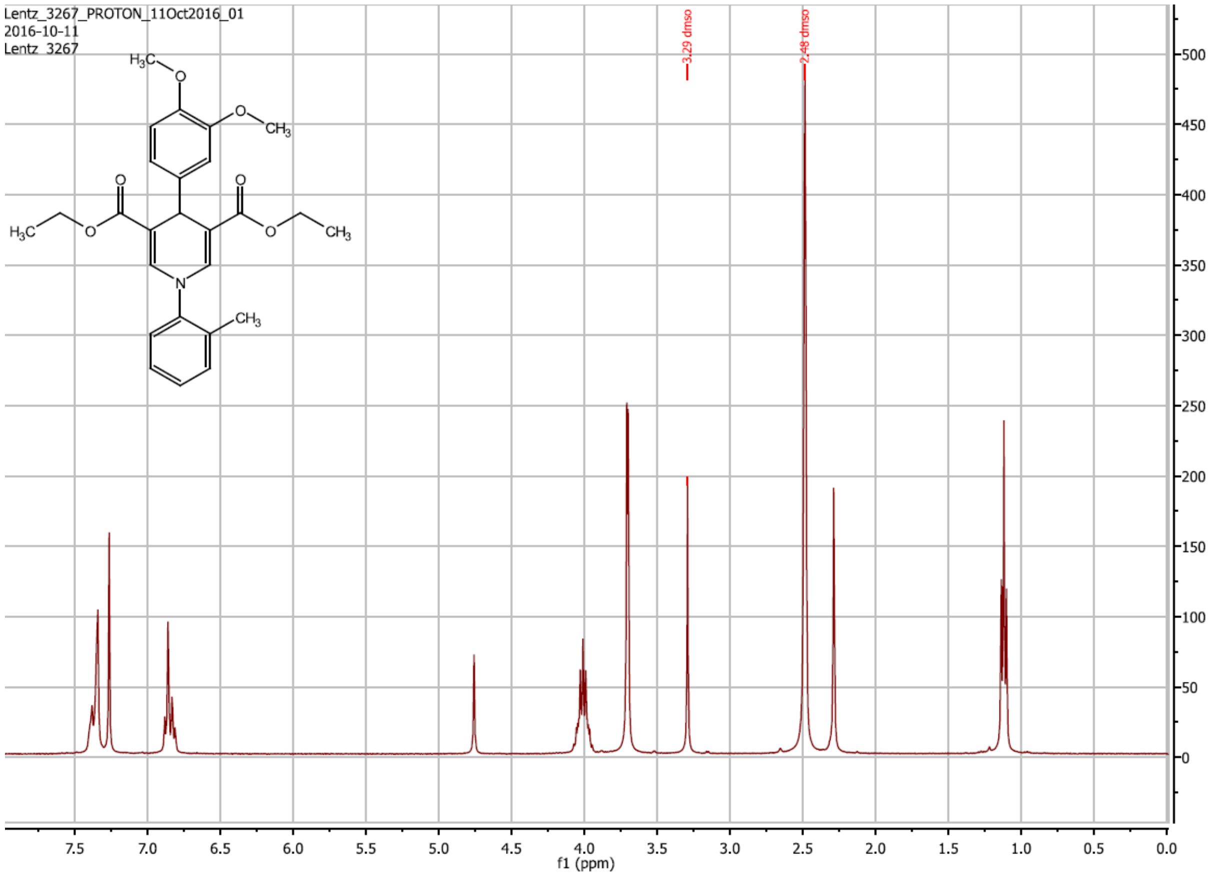

^{1}H NMR spectrum of compound **11**

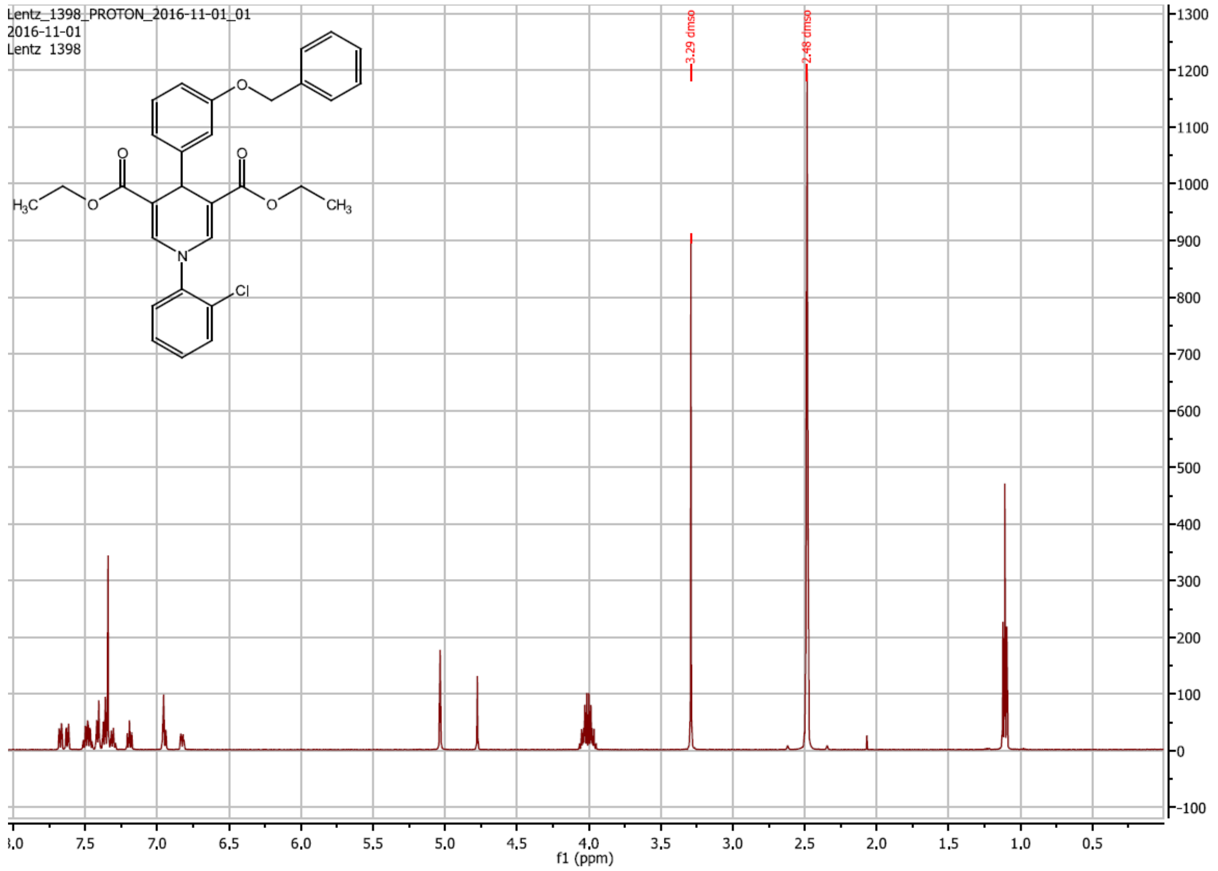

^{1}H NMR spectrum of compound **12**

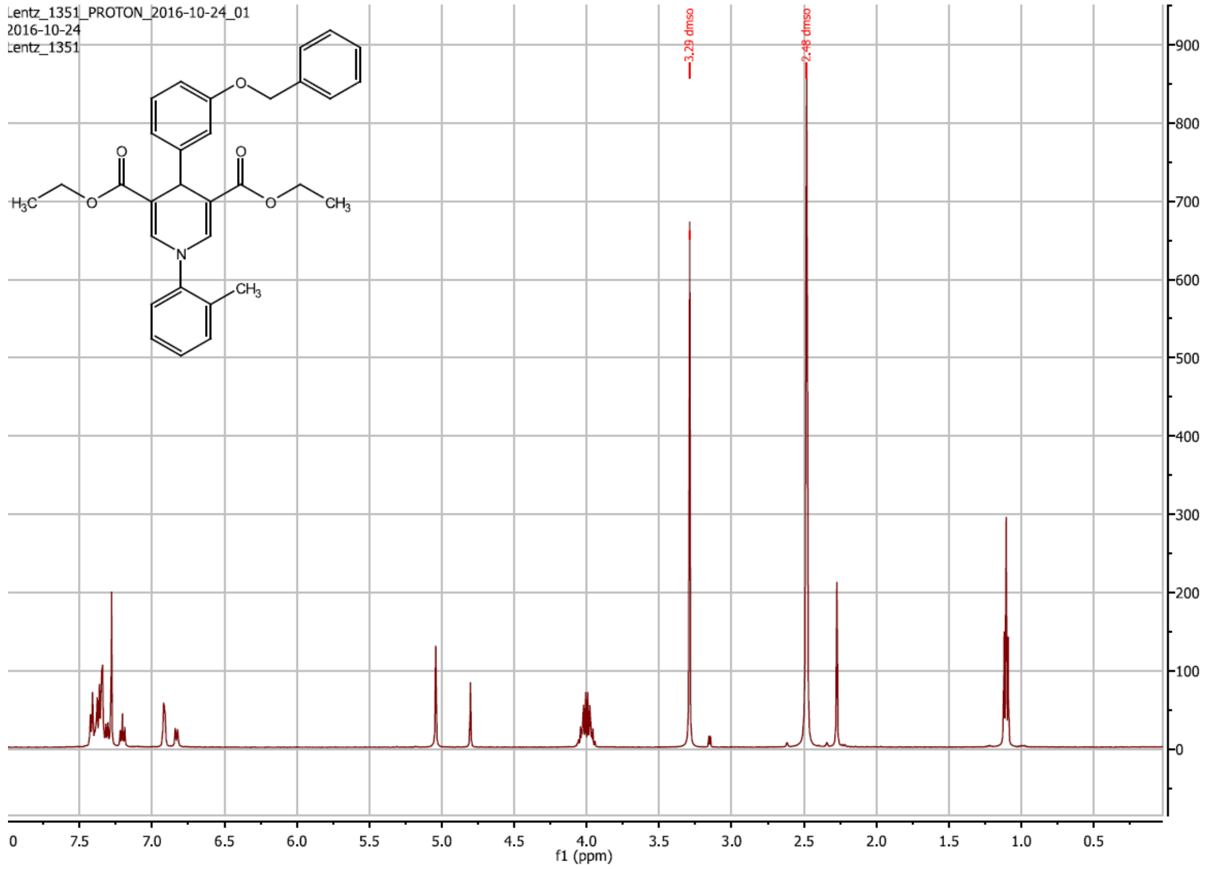

^{1}H NMR spectrum of compound **13**

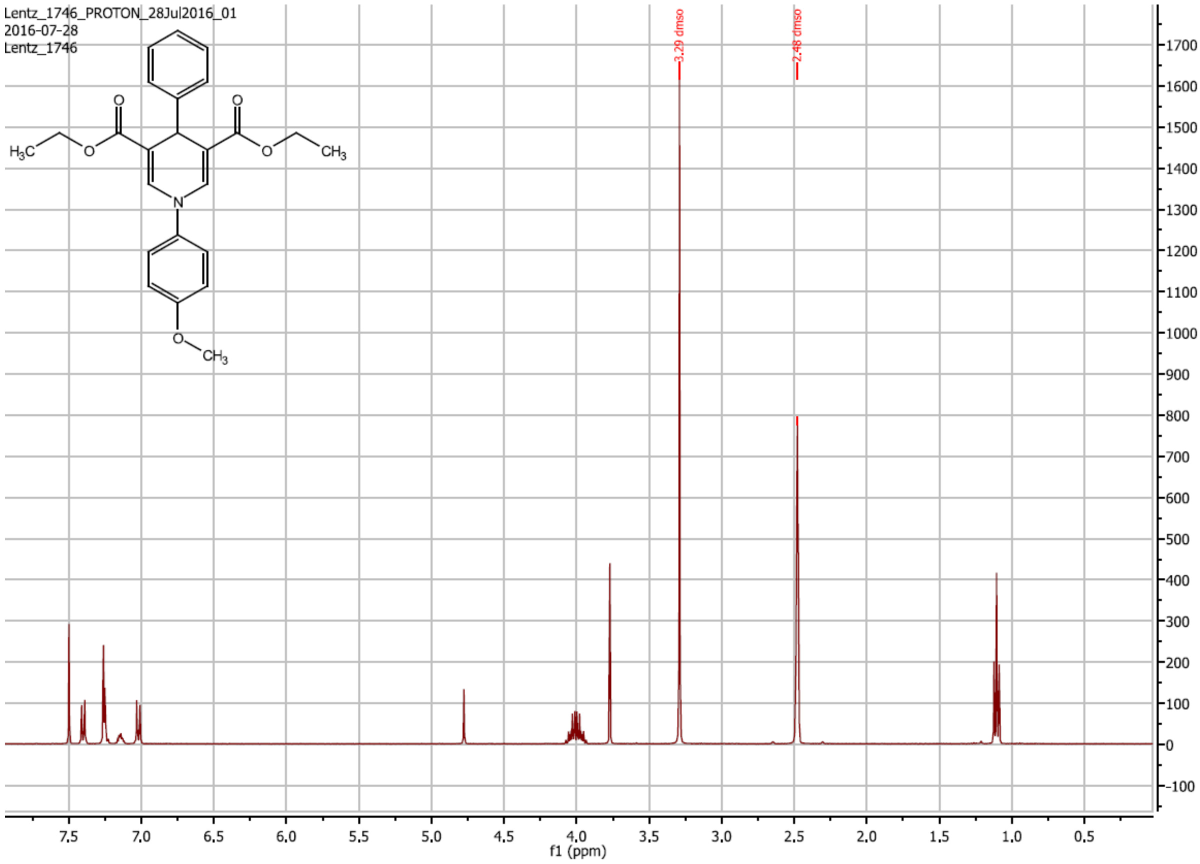

^{1}H NMR spectrum of compound **14**

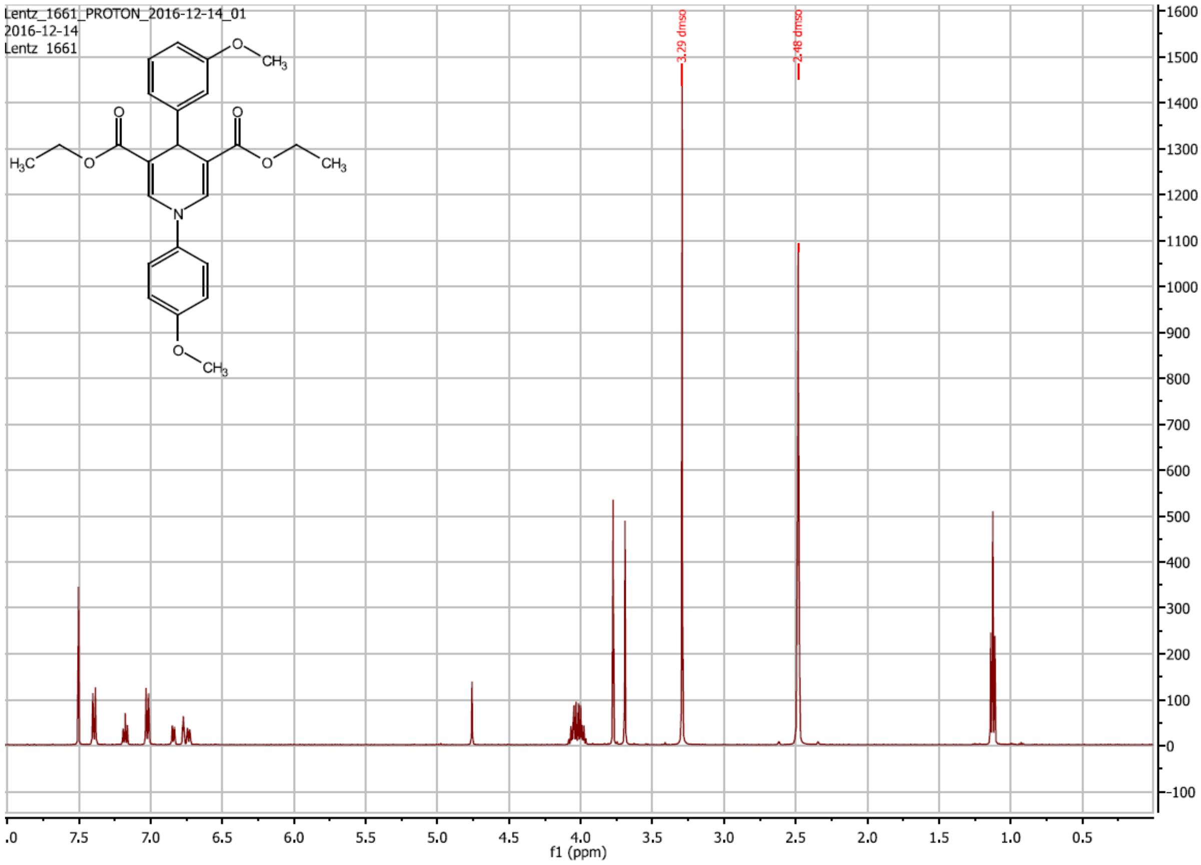

^{1}H NMR spectrum of compound **15**

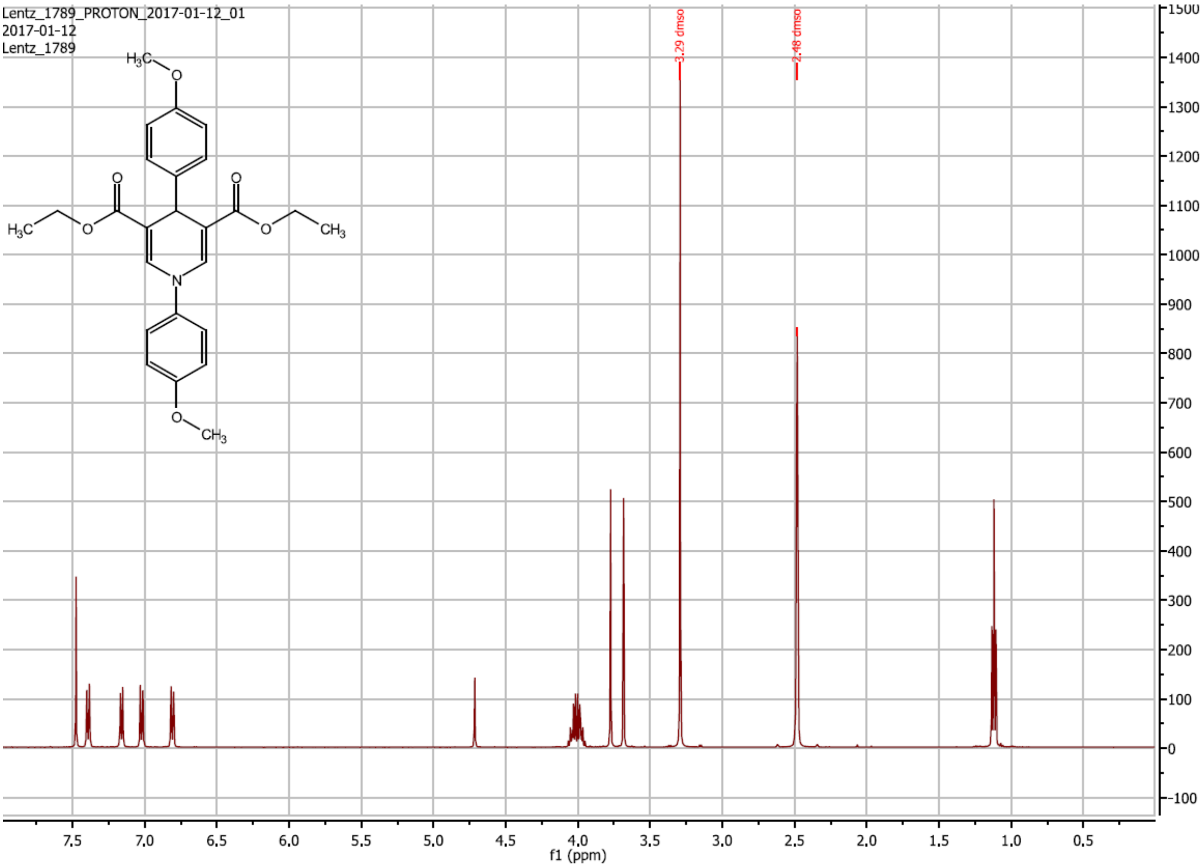

^{1}H NMR spectrum of compound **16**

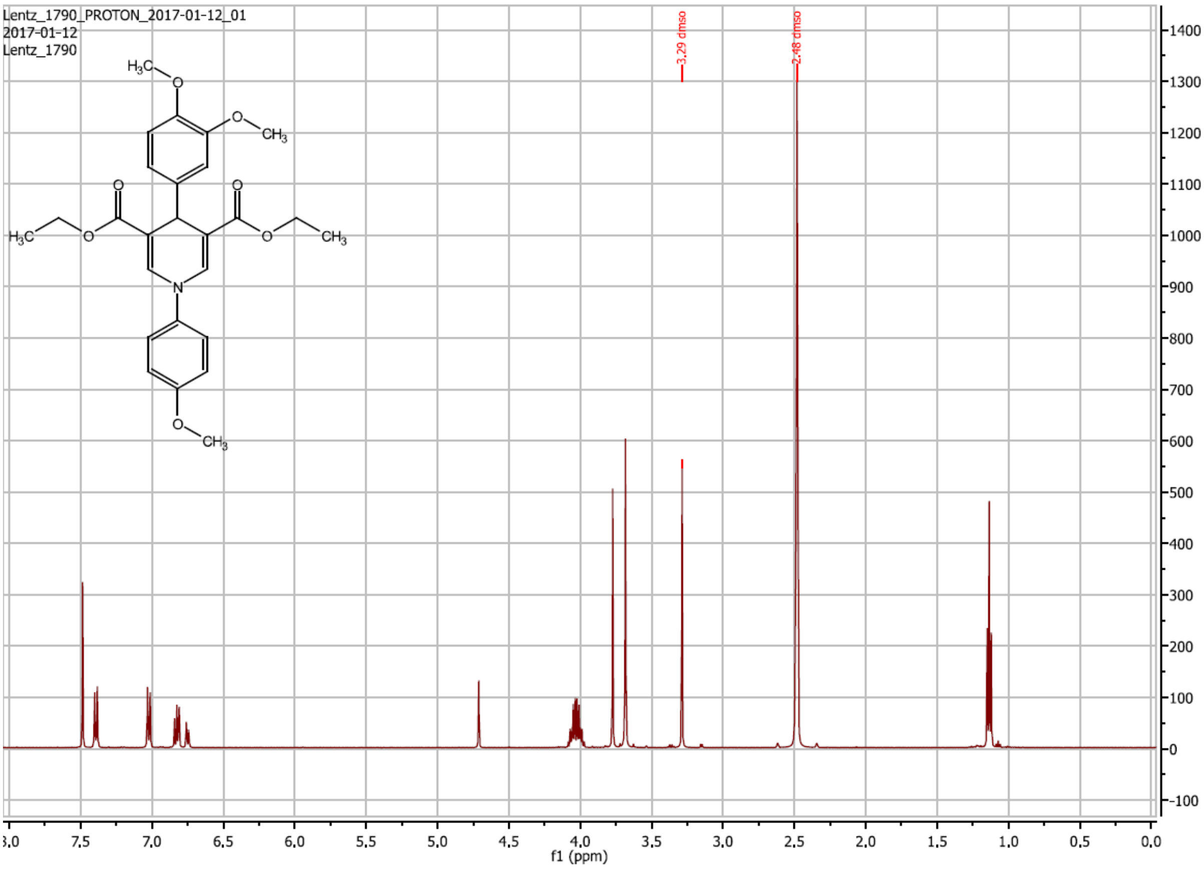

^{1}H NMR spectrum of compound **17**

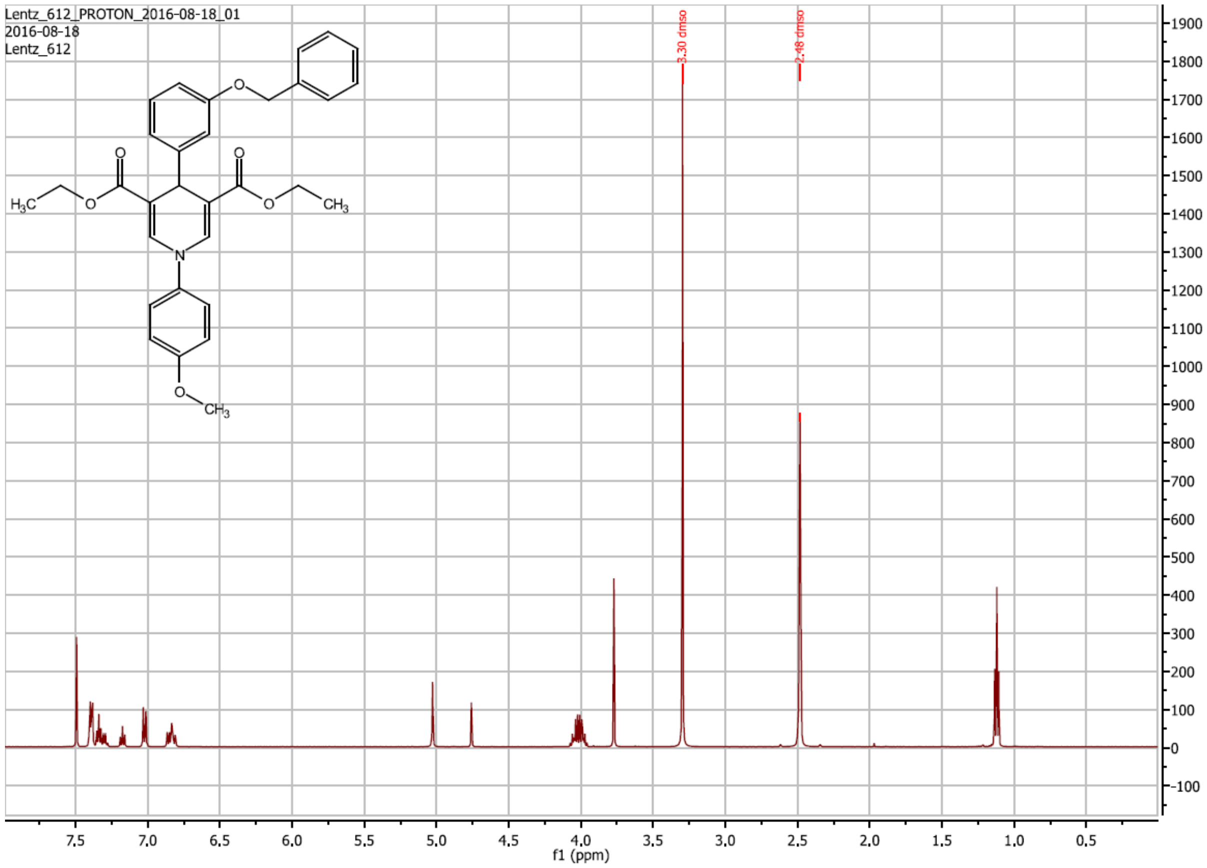

^{1}H NMR spectrum of compound **18**
